# Life satisfaction and chronic musculoskeletal pain at the baseline of ELSA-Brasil MSK

**DOI:** 10.1590/1980-549720250051

**Published:** 2025-11-03

**Authors:** Daniela Castelo Azevedo, Rosa Weiss Telles, Luciana Andrade Carneiro Machado, Sandhi Maria Barreto

**Affiliations:** I Universidade Federal de Minas Gerais, Faculty of Medicine - Belo Horizonte (MG), Brazil.; II Universidade Federal de Minas Gerais, Hospital das Clínicas/Empresa Brasileira de Serviços Hospitalares - Belo Horizonte (MG), Brazil.

**Keywords:** Psychological well-being, Personal satisfaction, Chronic pain, Musculoskeletal pain, Pain measurement, Bem-estar psicológico, Satisfação pessoal, Dor crônica, Dor musculoesquelética, Medição da dor

## Abstract

**Objective:**

The aim of this study was to investigate the association between life satisfaction and the presence and severity of chronic musculoskeletal pain (CMP).

**Methods:**

In this cross-sectional study, a total of 2,756 participants (mean age: 55.8 years, standard deviation [SD]=8.9 years) at the baseline of the Brazilian Longitudinal Study of Adult Health Musculoskeletal cohort (2012-2014) completed the Satisfaction with Life Scale and were assessed for CMP (duration>6 months) at neck, shoulders, upper back, elbows, lower back, wrists/hands, hips/thighs, knees, and ankles/feet. CMP phenotypes were identified based on measures that considered pain-related disability (non-disabling/disabling), pain demand for a healthcare professional (non-troublesome/troublesome), and body pain spreading according to the number of sites (0, 1-2, and ≥3, multisite) and the number of regions (upper limbs, lower limbs, and axial skeleton) affected (0, 1-2, 3, generalized). The association of life satisfaction with CMP and each CMP phenotype was investigated by binomial and multinomial logistic regression analyses adjusted for sociodemographic lifestyle and clinical confounders.

**Results:**

Greater life satisfaction was associated with lower chances of CMP (odds ratio [OR]=0.95; 95% confidence interval [CI] 0.94-0.97), as well as across all CMP phenotypes. The strength of this association was slightly greater for disabling CMP (OR=0.94; 95%CI 0.92-0.96) compared to non-disabling CMP (OR=0.97; 95%CI 0.95-0.99), and for troublesome CMP (OR=0.96; 95%CI 0.94-0.97) compared to non-troublesome CMP (OR=0.94; 95%CI 0.94-0.98). This association also held true when considering individuals experiencing multisite pain (OR=0.93; 95%CI 0.91-0.95) compared to those with pain at 1-2 sites (OR=0.97; 95%CI 0.95-0.99), and considering generalized pain (OR=0.93; 95%CI 0.90-0.96) compared to pain in 1-2 regions (OR=0.96; 95%CI 0.95-0.98).

**Conclusion:**

Greater life satisfaction seems to decrease the chances of experiencing any, and especially more severe, CMP.

## RESUMO

**Objetivo:**

Investigar a associação entre satisfação com a vida e presença e gravidade da dor musculoesquelética crônica (CMP).

**Métodos:**

Estudo transversal. No total, 2.756 participantes (média de idade 55,8, desvio padrão=8,9) na linha de base do Estudo Longitudinal de Saúde do Adulto musculoesquelético (ELSA-Brasil MSK) completaram a escala de satisfação com a vida e foram avaliados quanto à CMP (>6 meses) nas regiões: pescoço, ombros, partes superior e inferior das costas, cotovelos, punhos/mãos, quadris/coxa, joelhos, tornozelos/pés. Fenótipos de CMP foram identificados considerando incapacidade (não incapacitante/incapacitante), necessidade de médico (não problemática/problemática) e espalhamento com número de locais (0, 1-2 e ≥3-multissítio) e número de regiões (membros superiores, membros inferiores, esqueleto axial) afetadas (0, 1-2, 3-generalizada). A associação entre satisfação com a vida, CMP e seus fenótipos foi investigada por análises de regressão logística binomial e multinomial ajustadas para confundidores (sociodemográficos, estilo de vida, clínicos).

**Resultados:**

Maior satisfação com a vida foi associada a menores chances de CMP (*odds ratio* - OR=0,95; intervalo de confiança de 95% - IC95% 0,94-0,97) e seus fenótipos; a força da associação foi maior para dor incapacitante (OR=0,94; IC95% 0,92-0,96), em comparação com não incapacitante (OR=0,97; IC95% 0,95-0,99); e dor problemática (OR=0,96; IC95% 0,94-0,97), em comparação com não problemática (OR=0,94; IC95% 0,94-0,98). Com o espalhamento da dor: multissítio (OR=0,93; IC95% 0,91-0,95), em comparação com em 1-2 locais (OR=0,97; IC95% 0,95-0,99); e generalizada (OR=0,93; IC95% 0,90-0,96), em comparação com em 1-2 regiões (OR=0,96; IC95% 0,95-0,98).

**Conclusão:**

Maior satisfação com a vida parece diminuir as chances de CMP, especialmente a mais grave.

## INTRODUCTION

Musculoskeletal (MSK) disorders affect about one in every three people in the world^1^. Chronic musculoskeletal pain (CMP), a common symptom among individuals with MSK disorders, is responsible for the world’s largest disability burden^1^. Pain-associated disability is a recognized predictor of worse pain outcomes^2^ and premature mortality^3^. Chronic pain that exhibits a widespread distribution, assessed both by the number of pain sites and by the number of body regions affected, has also been shown to be associated with poorer quality of life^4^ and mortality^5^. The inadequate approach to chronic pain, without considering its biopsychosocial aspect in addition to the increase in opioid-prescribing practices in recent decades, has contributed to a concerning public health crisis in high-income countries^6^ ^,^ ^7^.

In the biopsychosocial context of chronic pain, psychological factors are considered important mediators of the association between chronic pain and negative health outcomes^8^ ^,^ ^9^. Although the link between chronic pain and negative psychological states (e.g., depression and anxiety) is well described in the literature^9^ ^,^ ^10^ ^,^ ^11^ ^,^ ^12^, its relationship with positive components has been poorly explored, even though these positive components can be therapeutic targets in the multidisciplinary treatment of chronic pain^13^.

Psychological well-being is related to the concept of living well, a combination of feeling good and functioning effectively^14^. It encompasses both eudaimonic and subjective (hedonic) well-being. Life satisfaction is one of the components of subjective well-being^15^ ^,^ ^16^, alongside positive affect and negative affect^16^. Life satisfaction refers to the cognitive evaluation of life, including positive and negative events in various domains (work, leisure, family life, community life, social life, and sexual life)^16^. Life satisfaction is a long-term construct: it deals not only with the actual emotional experiences (e.g., joy, affection, pride, sadness, and anger) but also with the cognitive evaluation of overall life and its salient domains^16^. One’s evaluation of one’s own life is determined by the aggregation of evaluations of positive and negative events of essential life domains (e.g., leisure life, work life, family life, community life, social life, and sexual life)^16^.

Few studies have investigated the association between life satisfaction and chronic pain^17^ ^,^ ^18^ ^,^ ^19^ ^,^ ^20^. Furthermore, to our knowledge, the Brazilian Longitudinal Study of Adult Health (ELSA-Brasil) Musculoskeletal cohort (ELSA-Brasil MSK) is the only study in a middle-income country that has collected information on both life satisfaction and multiple chronic pain phenotypes^21^.

The objective of this study was to investigate the association of life satisfaction with CMP phenotypes in ELSA-Brasil MSK. Our hypothesis is that individuals with higher levels of life satisfaction are less likely to have CMP, and that the magnitude of this association differs according to the severity of CMP.

## METHODS

### Study design and population

**Figure 1.**
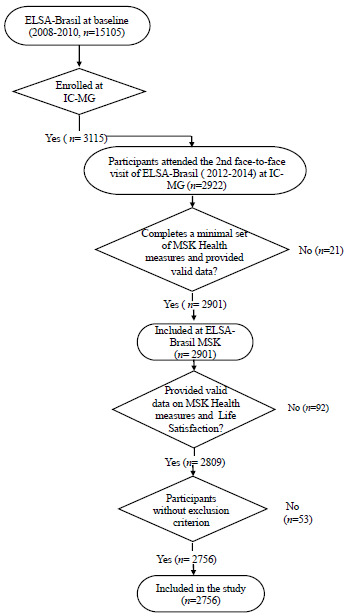
Flowchart of participants included in the study.

We conducted a cross-sectional analysis using data collected at baseline (2012-2014) of ELSA-Brasil MSK. The ELSA-Brasil MSK is an ancillary study to the multicenter cohort ELSA-Brasil, investigating the development, progression, and adverse health consequences of MSK disorders^22^. It comprises active/retired civil servants^22^. All servants enrolled at the Investigation Center of Minas Gerais (Universidade Federal de Minas Gerais - UFMG) and the Centro Federal de Educação Tecnológica de Minas Gerais - CEFET-MG), who attended the second face-to-face visit of the original cohort (2012-2014) were invited to participate. Those who completed a minimal set of MSK health measures and provided valid data were included (n=2,901). In the present study, we included ELSA-Brasil MSK participants who provided information on the response and explanatory variables (n=2,756, 95% of ELSA-Brasil MSK participants). We excluded individuals with a self-reported doctor’s diagnosis of inflammatory rheumatic diseases (rheumatoid arthritis, lupus erythematosus, rheumatism, arthrosis, and/or arthritis) and who also reported using antirheumatic drugs (abatacept, adalimumab, azathioprine, cyclosporine, hydroxychloroquine, leflunomide, methotrexate, and sulfasalazine). It was decided to exclude these diseases due to their particularities concerning pain, which is usually nociceptive pain, related to the lack of control over disease activity. The flowchart of participants included in the study is depicted in Figure 1.

ELSA-Brasil: Brazilian Longitudinal Study of Adult Health; MSK: musculoskeletal; IC-MG: Investigation Centre - Minas Gerais.

ELSA-Brasil and ELSA-Brasil MSK were approved by the ethics and research committee of the UFMG, Belo Horizonte, MG, Brazil (protocol COEP/UFMG, Ethics 186/06; CEP 1.160.939; CAAE 0186.1.203.000-06) and by the National Committee for Ethics in Research, Brazil (protocol 976/2006). All participants signed informed consent forms after being explained the nature and details of the study. This article has been written according to the Strengthening the Reporting of Observational Studies in Epidemiology (STROBE) guidelines.

### Assessment of chronic musculoskeletal pain

The assessment of CMP was performed using a standardized questionnaire and body diagram, based on the Nordic Musculoskeletal Questionnaire^23^, administered by trained and certified interviewers during face-to-face assessments.

The presence of pain at the following sites: neck, shoulders, upper back, elbows, lower back, wrists/hands (left/right), hips/thighs, knees (left/right), and ankles/feet was identified by the question “In the last 12 months, have you experienced pain, discomfort, or stiffness in the [site]?.” Those who responded positively were asked about chronic symptoms by the question “Did this problem that you had in the past 12 months last more than 6 months?.” Participants with pain at any of the investigated sites for >6 months were considered prevalent cases of CMP at any site, and those with symptoms of shorter duration or no CMP in the past 12 months were considered free of CMP. This definition of CMP lasting >6 months follows the recommendation of the International Association for the Study of Pain (IASP) expert group, which considers 6 months to be more suitable than 3 months for research purposes^24^.

Multiple severity phenotypes were investigated according to the presence of pain-associated disability, pain related to healthcare demand, and pain distribution pattern. Disabling CMP and pain related to healthcare demand (herein named troublesome CMP) were assessed in all participants presenting with CMP in at least one of the investigated sites and were considered present when the participant answered affirmatively to the following questions, respectively: “In the last 12 months, have you been prevented from doing your normal activities (e.g., work, domestic, and leisure activities) due to this (site) problem?” and “In the past 12 months, have you had pain, discomfort, or stiffness at (location) that caused you to see a healthcare professional (e.g., doctor, physical therapist)?.” The widespread pain distribution was assessed by counting the number of CMP sites (0, 1-2, ≥3, or multisite) and the number of body regions affected by CMP (0, 1-2, 3, or generalized). The three body regions considered were the upper limbs (shoulders, elbows, and/or hands), axial skeleton (neck, upper back, and/or lower back), and lower limbs (hips/thighs, knees, and/or ankles/foot)^24^ ^,^ ^25^. The evaluation of both the number of pain sites and the number of body regions is critical because it considers the sum of the number of painful sites (multisite pain) and the regional distribution of these sites (generalized pain), which may differ depending on the condition causing chronic pain^26^ ^,^ ^27^.

Five response variables were generated for the analysis: (1) CMP at any site (absent/present); (2) CMP according to disability (absent, non-disabling CMP, and disabling CMP); (3) CMP according to healthcare demand (absent, non-troublesome CMP, and troublesome CMP) and body spreading CMP (4) by number of sites (0, 1-2, ≥3, multisite); and (5) by number of regions (0, 1-2, 3, generalized).

### Assessment of life satisfaction

Life satisfaction was assessed by the Satisfaction with Life Scale^28^, already validated in Brazil^29^. This scale contains five items with statements about life satisfaction (“In general, my life is close to my ideal,” “The condition of my life are excellent,” I am satisfied with life,” “So far I have achieved the important things I want in life,” and “If I could live my life over, I would change almost nothing”), which are individually ranked on a 7-point Likert scale, i.e., 1: strongly disagree; 2: disagree; 3: slightly disagree; 4: neither agree nor disagree; 5: slightly agree; 6: agree; and 7: strongly agree. The total score ranges from five to 35 points, with higher scores indicating higher levels of life satisfaction.

### Assessment of confounders

Sociodemographic, lifestyle, and clinical characteristics were considered potential confounding factors for the relationship between life satisfaction and CMP. Basic sociodemographic data (age, sex, marital status, and level of education) were collected through standardized questionnaires during face-to-face assessments. The marital status question asked participants to identify their current situation. Participants who were married or living together were classified as “Married/United.” Separated or divorced were classified as “Separated/Divorced.” The education level was assessed by the educational background. Participants were grouped into the following education categories: (1) incomplete elementary school--never attended school or did not complete elementary school; (2) complete elementary school--those who completed elementary school but did not complete high school; (3) complete high school--those who have completed high school but did not attend university; and (4) complete higher education--those who completed university, higher education specialization, master’s, or doctoral degree.

Obesity was defined as a body mass index (BMI) ≥30 kg/m^2^. All participants were weighed on electronic scales (Toledo^®^, capacity 200 kg), and their height was measured on a stadiometer with a millimeter scale (SECA^®^, SE-216).

The level of leisure-time physical activity was assessed by the International Physical Activity Questionnaire (IPAQ 2005) and categorized as follows: (1) low, when participants reported no physical activity or practicing less than the other categories; (2) moderate, when the practice was 3 or more days of vigorous activity for at least 20 min/day, 5 or more days of moderate activity and/or walking for at least 30 min/day, or 5 or more days of any combination of walking, moderate, or vigorous intensity activities that reach; (3) high, when the patient reported vigorous activity for at least 3 days and accumulated at least 1500 MET-min/week or 7 or more days of any combination of walking, moderate, or vigorous activities, accumulating at least 3,000 MET-min/week^30^.

Smoking was considered present if the participant reported having smoked at least 100 cigarettes in their lifetime and responded positively to the question about current smoking.

Both depression and the use of antidepressants were considered as confounders because some antidepressants are used to treat chronic pain regardless of the presence of depression^31^. Depression in the past 7 days was assessed by section G of the Clinical Interview Schedule-Revised Version (CIS-R), a version adapted to Brazilian Portuguese and validated for use in the ELSA-Brasil sample, after application of a specific algorithm^32^. The use of antidepressants was assessed by asking the participant about the medications they were continuously using in the last 15 days and checking their prescriptions and medication boxes on the day of the interview. Subsequently, drug classes were identified and assembled using the World Health Organization’s Anatomical Therapeutic Chemical Classification system (WHO ATC)^33^.

### Statistical analysis

Descriptive analyses used frequencies and percentages (%) for categorical variables and means and standard deviations (SDs) for continuous variables.

The association of life satisfaction with any CMP (absent/present) was investigated using binomial logistic regression. Associations between life satisfaction and each investigated pain severity phenotype (CMP according to disability, CMP according to healthcare demand, CMP according to the number of sites affected, and CMP according to the number of regions affected) were estimated separately by multinomial logistic regressions, always considering the absence of CMP as the reference category.

All analyses followed the steps described below. Model 0 (crude model) tested the association of life satisfaction with CMP and each CMP pain severity phenotype without any adjustments. After estimating the crude association (Model 0), the adjustment for age, sex, education, and marital status was made (Model 1); then, obesity, physical activity, smoking, and use of antidepressants were added to model 1 (Model 2); finally, depression was added to model 2 (Model 3). The magnitudes of the associations between life satisfaction and CMP and CMP severity phenotype were estimated by the odds ratio (OR) (and its 95% confidence intervals [95% CIs]).

Analyses were performed using STATA (version 14.0, StataCorp LP, College Station, USA), with 95% confidence level (α=5%).

### Data availability statement

Data available upon request.

## RESULTS

The total sample comprised 2,756 participants (Figure 1) with a mean age of 55.8 years (SD: 8.9), with a slight female predominance (52.5%). The sociodemographic and clinical characteristics of the included participants, as well as those belonging to the subgroup with no CMP, CMP at any site, and CMP prevalence, are described in Table 1. Most of the individuals included in the study were married/united, had a higher level of education, and reported low levels of leisure-time physical activity. Overall, CMP was more frequent in women, in the 55-64 years’ age group, in obese participants, and in those with a diagnosis of depression (Table 1).

**Table 1.** Distribution of the study population and the prevalence of chronic musculoskeletal pain (CMP) according to sociodemographic and health characteristics at the baseline of ELSA-Brasil MSK (2012-2014).

Sociodemographic and health characteristics	Total sample^I^ n=2,756 (%)	Participants without CMP n=1,262 (%)	CMP participants n=1,494 (%)	Prevalence of CMP at any site^J^ n=1,494 (%)
Sex				
Female	1,447 (52.0)	528 (41.9)	919 (61.5)	63.5
Male	1,309 (48.0)	734 (58.1)	575 (38.5)	44.0
Age group^a^				
35-44	284 (10.3)	156 (12.4)	128 (8.6)	45.1
45-54	1,005 (36.5)	468 (37.1)	567 (35.9)	53.4
55-64	982 (35.7)	423 (33.5)	559 (37.4)	57.0
65-79	484 (17.5)	214 (17.0)	270 (18.1)	55.0
Level of education^b^				
Completed higher education	1,835 (66.6)	867 (68.8)	968 (64.9)	52.8
Completed high school	695 (25.3)	298 (23.6)	397 (26.6)	57.1
Completed elementary school	120 (4.4)	48 (3.8)	72 (4.8)	60.0
Did not complete elementary school	103 (3.7)	48 (3.8)	55 (3.7)	53.4
Marital status^c^				
Married/united	1,456 (52.8)	703 (55.8)	753 (50.4)	51.7
Separated/divorced	317 (11.5)	151 (11.9)	166 (11.1)	52.0
Single	160 (5.8)	65 (5.2)	95 (6.4)	59.4
Widower	256 (9.4)	109 (8.6)	147(9.8)	57.0
Other	566 (20.5)	233 (18.5)	333 (22.3)	59.0
Obesity (BMI≥30 kg/m^2^)^d^				
No	2,140 (77.6)	1,034 (82.0)	1,106 (74.0)	52.0
Yes	615 (22.4)	227 (18.0)	388 (26.0)	63.0
Leisure-time physical activity^e^				
Low	1945 (70.6)	834 (66.1)	1,111 (74.4)	57.1
Moderate	580 (21.0)	310 (24.6)	270 (18.1)	46.6
High	230 (8.4)	117 (9.3)	113 (7.5)	50.0
Smoking^f^				
No	2,490 (90.0)	1,143 (90.6)	1,347 (90.2)	54.0
Yes	265 (10.0)	118 (9.4)	147 (9.8)	56.0
Use of antidepressants^g^				
No	2,358 (85.6)	1,133 (90.1)	1,255 (82.4)	52.0
Yes	385 (14.4)	124 (9.9)	261 (17.6)	67.8
Depression^h^				
No	2,608 (95.0)	1,226 (97.2)	1,382 (92.5)	53.0
Yes	147 (5.0)	35 (2.8)	112 (7.5)	76.0

CMP: chronic musculoskeletal pain; BMI: body mass index; Data presented as frequency and percentages for valid cases only. Frequency of missing data ^a^1 ^b^3; ^c^3 ^d^1; ^e^1 ^f^1; ^g^13 ^h^1; ^I^distribution of characteristics of the study population; ^J^prevalence of CMP: proportion of CMP participants in each category of the sociodemographic and health characteristics variables.

Almost all study participants (94.9%) reported MSK pain in the past 12 months in at least one location, and in just over half (54.2%), the pain was characterized as chronic (≥6 months). Table 2 details the prevalence of CMP pain severity phenotypes.

**Table 2.** Prevalence of CMP pain severity phenotype at the baseline of ELSA-Brasil MSK (2012-2014).

CMP pain severity phenotypes	Prevalence n (%)
CMP according to disability^a^	
CMP non-disabling	861 (31.2)
CMP disabling	633 (22.9)
CMP related to health demand^b^	
CMP non-troublesome	490 (17.7)
CMP troublesome	1,004 (36.4)
CMP according to the number of sites affected^c,*^	
1-2 sites	975 (35.5)
≥3 (multisite)	508 (18.5)
CMP according to the number of regions affected^d,**^	
1-2 regions	1,215 (44.2)
3 (generalized)	274 (9.7)

CMP: chronic musculoskeletal pain. Frequency of missing data ^*^11; ^**^5; ^a^pain that prevented normal activities; ^b^pain related to health demand; ^c^sites assessed: shoulders, elbows, wrist/hands (left/right), neck, upper back, lower back, hips/thighs, knees (left/right), and ankles/feet; ^d^three regions assessed: upper limbs (shoulders, elbows, and/or wrist/hands), axial skeleton (neck, upper back, and/or lower back), and lower limbs (hips/thighs, knees, and/or ankles/feet).

The most frequent sites of CMP reported among the nine sites investigated were the knee (21.7%), followed by the lower back (18.2%), shoulders (17.6%), ankles/feet (16.4%), neck (13.5%), wrists/hands (12.7%), upper back (10.8%), hips/thighs (9.9%), and elbows (5,4%). The prevalence of CMP according to regions was highest in the lower limbs (35.0%), followed by pain in the axial skeleton (29.1%) and upper limbs (27.0%).

Table 3 describes the results of the crude model (Model 0) and multivariable models (Models 1-3) regarding the association of life satisfaction with CMP phenotypes at ELSA-Brasil MSK. Table 3 shows that, after all adjustments (Model 3), each one-point increase in the life satisfaction scale reduced the odds of reporting CMP at any site by 5% (adjusted OR=0.95; 95%CI 0.94-0.97), the odds of disabling CMP decreased by 6% (adjusted OR=0.94; 95%CI 0.92-0.96), and the odds of troublesome CMP decreased by 4% (adjusted OR=0.96; 95%CI 0.94-0.97). The results from Model 3 display that life satisfaction was also inversely associated with both measures of widespread CMP, all compared with no CMP, with slightly stronger associations observed for multisite pain (OR=0.93; 95%CI 0.91-0.95) compared to pain at 1-2 sites (OR=0.97; 95%CI 0.95-0.99), and for generalized CMP (OR=0.93; 95%CI 0.90-0.96) compared to pain in 1-2 regions (OR=0.96; 95%CI 0.95-0.98).

**Table 3.** Association of life satisfaction with distinct chronic musculoskeletal pain (CMP) severity phenotypes at the baseline of ELSA-Brasil MSK (2012-2014).

CMP phenotypes		Crude OR	Adjusted OR		
		Model 0 OR (95%CI)	Model 1 OR (95%CI)	Model 2 OR (95%CI)	Model 3 OR (95%CI)
CMP at any site		0.95 (0.94-0.97)	0.95 (0.94-0.97)	0.95 (0.93-0.97)	0.95 (0.94-0.97)
CMP according to disability^a^	No CMP	ref	ref	ref	ref
	Non-disabling	0.97 (0.95-0.99)	0.96 (0.95-0.98)	0.97 (0.95-0.98)	0,97 (0.95-0.99)
	Disabling	0.93 (0.91-0.95)	0.93 (0.91-0.95)	0.94 (0.92-0.95)	0.94 (0.92-0.96)
CMP according to healthcare demand^b^	No CMP	ref	ref	ref	ref
	Non-troublesome	0.96 (0.94-0.98)	0.95 (0.93-0.97)	0.96 (0.94-0.98)	0.96 (0.94-0.98)
	Troublesome	0.95 (0.94-0.97)	0.95 (0.93-0.96)	0.95 (0.93-0.97)	0.96 (0.94-0.97)
CMP according to the number of sites affected^c^	No CMP	ref.	ref.	ref.	ref.
	1-2	0.97 (0.95-0.99)	0.97 (0.95-0.99)	0.97 (0.95-0.99)	0.97 (0.95-0.99)
	≥3 (multisite)	0.92 (0.90-0.94)	0.92 (0.90-0.94)	0.92 (0.90-0.94)	0.93 (0.91-0.95)
CMP according to the number of regions affected^d^	No CMP	ref.	ref.	ref.	ref.
	1-2	0.96 (0.95-0.98)	0.96 (0.94-0.97)	0.96 (0.94-0.98)	0.96 (0.95-0.98)
	3 (generalized)	0.92 (0.90-0.94)	0.92 (0.89-0.94)	0.92 (0.89-0.94)	0.93 (0.90-0.96)

CMP: chronic musculoskeletal pain; OR: odds ratio; 95%CI: 95% confidence interval; ^a^pain that prevented normal activities; ^b^pain related to healthcare demand; ^c^sites assessed: shoulders, elbows, wrist/hands (left/right), neck, upper back, lower back, hips/thighs, knees (left/right), and ankles/feet; ^d^three regions assessed: upper limbs (shoulders, elbows, and/or hands), axial skeleton (neck, upper back, and/or lower back), and lower limbs (hips/thighs, knees, and/or ankles/feet); model 0: no adjustment; model 1: model 0 plus age, sex, education, and marital status; model 2: model 1 plus obesity, physical activity, smoking, and use of antidepressants; model 3: model 2 plus depression.

## DISCUSSION

In this cohort of middle-aged and older civil servants, greater life satisfaction was inversely associated with CMP at any site as well as with all CMP pain severity phenotypes, i.e., pain associated with disability, pain related to healthcare demand, and widespread pain (multisite and generalized pain). The magnitudes of association were greater for multisite pain, generalized pain, and disabling pain. The association was consistent, remaining after adjustments, including for depression and antidepressant use.

Results support the evidence that life satisfaction, which belongs to subjective well-being, does not reflect the simple absence of negative psychological states^34^ ^,^ ^35^. Studies evaluating the relationship between psychological factors and CMP mainly consider the negative aspect of well-being (e.g., depression and anxiety)^36^. The vast majority of studies included in a review of systematic reviews and meta-analyses of longitudinal studies (249,657 participants) on psychological factors associated with the onset and persistence of MSK pain mainly evaluated negative psychological states^36^.

A few other studies have also shown a relationship between psychological well-being, such as life satisfaction, and chronic pain in general, including CMP and conditions associated with CMP, like arthritis. A 9-year follow-up analysis of an European cohort of 10,530 individuals, aged 50 years and older, showed that better psychological well-being, as measured by the Control, Autonomy, Self-realization, and Pleasure-12 (CASP-12) instrument (which assesses the psychological dimensions of control, autonomy, self-realization, and pleasure), was associated with a lower incidence of self-reported medical diagnosis of arthritis, a common cause of CMP^37^. In a sample of 232 patients from a physical medicine and rehabilitation service in Norway, pain intensity was found to be inversely associated with overall life satisfaction, as measured by a different instrument, the LiSat-9 scale. This instrument assesses life satisfaction both in general and across specific domains, namely self-care, contact with friends, vocational situation, family life, relationship with partner, financial situation, leisure, and sexual life^19^. In that study, widespread pain, assessed by the number of painful sites, was associated only with the domains of financial and vocational life satisfaction^19^. However, the study sample of this latter study was small, and unlike the present research, the reference category consisted entirely of patients with pain.

Another study has also examined whether chronic pain is associated with worsening subjective well-being over time, a direction of association opposite to the hypothesis assumed in this work (life satisfaction is an explanatory variable)^38^. In a cohort of 437 middle-aged adults with chronic pain, pain was found to be associated with lower life satisfaction^38^. However, longitudinal analysis showed that the presence of chronic pain did not predict a decrease in subjective well-being over 10 years^38^. We believe that the reason for this finding regarding life satisfaction might be the fact that life satisfaction predicts pain, and not the other way around. Life satisfaction is a cognitive evaluation of life across multiple domains and may not be influenced by momentary suffering experiences, such as pain. Life satisfaction represents a comprehensive, summative evaluation of both positive and negative events in key life domains, and it is a long-term, enduring assessment^39^. This is why we have hypothesized that life satisfaction would be associated with CMP in this study and not the contrary.

We believe that the association of greater life satisfaction with lower chances of CMP could be due to the interference of life satisfaction with the central sensitization process. Life satisfaction is one of the components of subjective well-being^15^. The other two components are affective: (1) positive affect (joy, affection, pride) and feelings of happiness and (2) negative affect (sadness, anger, blame, anxiety) and feelings of sadness^16^. Highest levels of subjective well-being might protect individuals from chronicity, associated disability, and “spreadness” of CMP by attenuating the activation of the hypothalamic-pituitary-adrenal system and sympathetic nervous system by stress^40^. The process triggered by stress would be partially inhibited, with less release of pro-inflammatory cytokines and absence of changes in peripheral nociceptors that contribute to central sensitization and consequent chronic pain^41^. Such a hypothesis has been explored with positive affect. A study showed that positive affect was associated with lower levels of interleukin-6 (IL-6) and C-reactive protein in women, regardless of age, BMI, smoking, and depressed mood, in accordance with the pathophysiological hypothesis presented earlier^40^.

The inverse gradient of the association between life satisfaction and disabling CMP suggests that life satisfaction might contribute to pain coping strategies by reducing associated disability^42^ since higher life satisfaction decreases the chance of disabling CMP with a slightly more significant magnitude of association than non-disabling CMP, both in relation to the absence of CMP.

Treatments for CMP should consider its psychological aspect^43^ ^,^ ^44^. An inadequate therapeutic approach can overload the healthcare system and increase the use of opioids^45^. Besides being ineffective in this type of pain^46^, opioids can lead to serious adverse events, such as respiratory depression and dependence, with great individual and population impacts^45^.

Psychological well-being, along with social and economic indicators, represents an indicator of quality of life for individuals and societies. Subjective well-being is complementary to the objective indicators of well-being (health, pollution, and income)^39^. Improving objective indicators of well-being can help but might not be sufficient to enhance life satisfaction substantially^39^. In this sense, the inverse relationship of life satisfaction with chronicity and severity of CMP can support the use of positive psychological interventions (PPIs) as a complementary strategy to treating MSK pain. More studies are needed to evaluate the effectiveness of such interventions.

The present study gathered a considerable sample of participants and was conducted at the baseline of the ELSA-Brasil MSK cohort, a study that uses strict protocols and validated questionnaires. To our knowledge, this is the first study in a community sample that shows an association between life satisfaction and the severity phenotypes of CMP, as assessed by the presence of associated disability, healthcare demand, and widespread pain. However, due to its cross-sectional design, no causal relationship can be established, and the possibility that CMP and its severity decrease life satisfaction cannot be ruled out. Since the study is part of a cohort, the follow-up of the participants will allow for testing the hypothesis that life satisfaction may be a protective factor for the development and worsening of CMP.

In conclusion, the present study reveals that greater life satisfaction is inversely associated with CMP, particularly severe pain phenotypes. This result reinforces the importance of further investigating the relationship between CMP and life satisfaction longitudinally. This study sheds new light on the treatment perspective of CMP, emphasizing the importance of the positive aspect of psychological state.
